# Structural Perspective on Revealing and Altering Molecular Functions of Genetic Variants Linked with Diseases

**DOI:** 10.3390/ijms20030548

**Published:** 2019-01-28

**Authors:** Yunhui Peng, Emil Alexov, Sankar Basu

**Affiliations:** Department of Physics and Astronomy, Clemson University, Clemson, SC 29634, USA; yunhuip@g.clemson.edu (Y.P.); ealexov@g.clemson.edu (E.A.)

**Keywords:** mutations, disease-causing mutations, polymorphism, folding free energy change, binding free energy change, drug discovery, in silico screening

## Abstract

Structural information of biological macromolecules is crucial and necessary to deliver predictions about the effects of mutations—whether polymorphic or deleterious (i.e., disease causing), wherein, thermodynamic parameters, namely, folding and binding free energies potentially serve as effective biomarkers. It may be emphasized that the effect of a mutation depends on various factors, including the type of protein (globular, membrane or intrinsically disordered protein) and the structural context in which it occurs. Such information may positively aid drug-design. Furthermore, due to the intrinsic plasticity of proteins, even mutations involving radical change of the structural and physico–chemical properties of the amino acids (native vs. mutant) can still have minimal effects on protein thermodynamics. However, if a mutation causes significant perturbation by either folding or binding free energies, it is quite likely to be deleterious. Mitigating such effects is a promising alternative to the traditional approaches of designing inhibitors. This can be done by structure-based in silico screening of small molecules for which binding to the dysfunctional protein restores its wild type thermodynamics. In this review we emphasize the effects of mutations on two important biophysical properties, stability and binding affinity, and how structures can be used for structure-based drug design to mitigate the effects of disease-causing variants on the above biophysical properties.

## 1. Effect of Mutations on Stability and Binding

The study of the effect of amino acid mutations within proteins has been a traditional chapter in protein science. Earlier studies were applied site-directed mutagenesis to assess the importance of an amino acid for stability and function of the corresponding protein. Nowadays, the focus has shifted to understanding the effects caused by genetic variants, namely, non-synonymous single nucleoside polymorphisms (nsSNP), with respect to disease predisposition. The phage-T4 lysozyme, for example, has served as one of the most well-studied systems with regard to mutations [1]. These studies were facilitated by the availability of X-ray structures (native and mutants) allowing for structural investigations of the effects on protein packing, stability, and activity. At the same time, lysozyme mutants in human have also been studied to characterize the molecular mechanism of diseases, such as hereditary systemic amyloidosis [2]. Similarly, the barnase–barstar protein-inhibitor complex was subjected to extensive mutagenesis to reveal the role of various residues on binding affinity [3,4,5]. This high-resolution complex has served as a model system to study protein–protein recognition by single and double mutant cycles [4,5]. Such studies have also served to rationalize optimization theories in the binding energetics [5,6] generally applicable to protein–protein recognition. Recent advances in the study of genetic (DNA) variants in the same system have also explored their influence in the manifestation of differential immunogenicity, and this very property has then been applied in bio-therapeutics, for example, by constructing heterodimeric barnase–barstar DNA vaccine molecules [7], ground-breaking in the development of novel DNA vaccines. Effectively, numerous works in molecular biophysics were and are focusing on understanding the effects of mutations on protein stability and binding. Below we review the relevant concepts and works associated with the two most fundamental biophysical events in protein science, folding and binding. 

**Figure 1:**
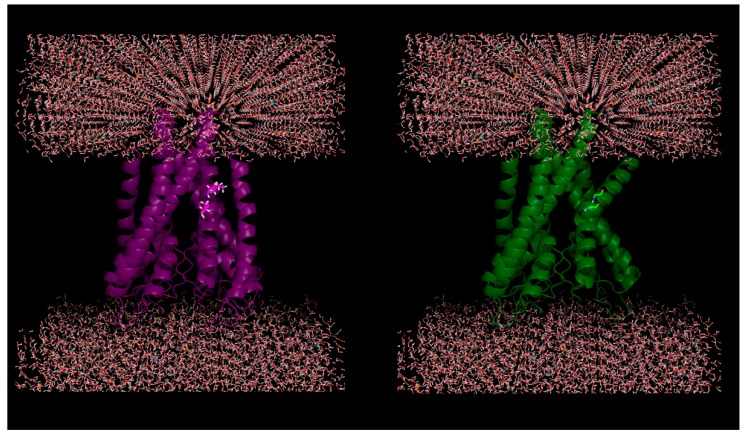
Plausible effect of mutations in membrane proteins: Helical kink is introduced due to in silico mutations of two successive residues (100-Ile, 101-Thr) to glycine (helix breaker) in a KcsA potassium channel protein (PDB ID: 1J95).

To begin with the effects of amino acid substitutions on protein folding, we emphasize that the same substitution may have different effects when occurring in globular, membrane or intrinsically disordered proteins. It is perhaps good to reiterate the fact that globular proteins are characterized by the presence of densely packed interiors (hydrophobic core) with packing densities (0.7 to 0.8) resembling that of crystalline solids [8] and, that, interior packing is known to be one of the most dominant forces in protein folding [9], also related to the stability, dynamics and the de novo design of the foldable globules. The dense interior packing within globular proteins is known to be achieved by a nucleation–condensation of ‘packing motifs’ [10], concomitant to the rapid collapse of hydrophobic residues in an aqueous environment. On the other hand, helix packing in integral membrane proteins [11] inserted within the lipid bilayer does not involve the ‘hydrophobic effect’ and yet, scales to an equivalent magnitude of packing to that of the globular proteins [12]. The polar vs. hydrophobic environment presented in the two cases demands differential amino acid compositions in the two types of proteins to achieve an equivalent degree of packing in both. Interestingly, small hydrophobic (Gly, Ala) [11,12] and small hydroxyl-containing (Ser, Thr) [12] amino acids have been found to contribute the most in tight packing of helices in membrane proteins as opposed to large hydrophobic and aromatic residues [10,13] in globular protein interiors. Apart from the tight packing of helices, membrane proteins are also known to involve a distinct pattern of charges [14,15] embedded in their sequence to remain stable and active within the amphiphilic lipid bilayer. In dramatic contrast, in the case of intrinsically disordered proteins (IDPs), the interior packing is practically negligible [16], since (unlike globular proteins) the few hydrophobic residues in them are so placed that it forbids the possibility of a hydrophobic collapse to attain a stable fold with a well-packed core. This, in fact, enables them to retain their characteristic disorder or dynamic flexibility using existing conformational ensembles rather than a single stably folded global minima structure similar to either globular [9] or membrane proteins [17]. The major component in retaining this dynamic flexibility in IDPs is electrostatic interactions [18] involving hydrogen bonds, salt-bridges, charge–dipole, and dipole–dipole interactions. Hence, when subjected to mutational studies, the sites to perform the mutations are chosen based on the knowledge-based prediction of the expected differential effect in folding, stability, and dynamics for the three major class of proteins. For example, salt-bridge mutations have served to constitute one of the prime chapters in understanding the *modus operandi* in IDPs [19,20] while the study of hydrophobic core mutations has traditionally served to probe interior packing within globular proteins [21], which will be discussed in more detail in the next section. Electrostatics also serves as an indispensable component in the folding and stability of globular proteins [22]. For membrane proteins, mutations have been chosen mostly based on structure–function relationships [23], such as oligomerization [24], thermostability [25], etc., involving both packing and electrostatics. There have also been instances of strategic point mutations (e.g., involving proline and/or glycine the well-known helix-breakers) introducing kinks (Figure 1) and wedging on transmembrane helix–helix interfaces [26]. Apart from the specific emphasis on individual structure–functional attributes of these different classes of proteins, mutational studies have also been attempted as a mean to trace their evolutionary origin (or common ancestor), particularly relevant in the context of the ‘globular-disordered interface’ [27,28] in proteins. 

## 2. Mutation and its Compensation: Structural Plasticity and Conformational Relaxation

**Figure 2:**
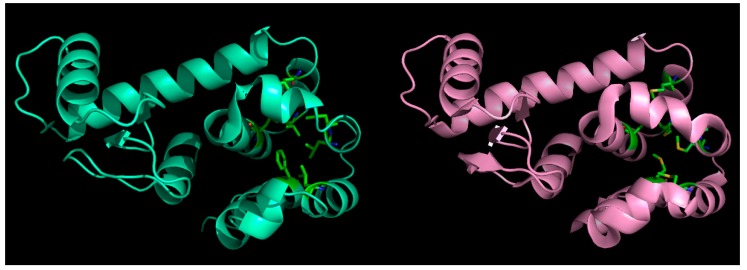
Effect of mutations in globular proteins: seven core (hydrophobic) residues mutated to methionine (left panel: native, right: mutant) in phage T4-lysozyme and yet, the fold is preserved without almost any marked distortions. This happens because of ‘structural relaxation’ in proteins due to their inherent conformational plasticity (adaptability to changes).

Plasticity in the context of protein conformations [29,30,31] describes their adaptability in response to applied external forces (for example, by introducing mutations). This is a key physical property for protein dynamics, wherein, it serves to facilitate protein evolution and other protein functions, such as allostery and self-assembly [32]. Structural studies have further shown how conformational relaxation of both main- and side-chain atoms could compensate the deleterious effects of mutations, thereby, preserving the overall fold [33,34]. The random mutation of the 12 out of 13 core residues of ribonuclease barnase is an example where 23% of the mutants retained their enzymatic activity in vivo [35]. Other similar studies followed, and the idea of introducing strategic multiple mutations was eventually extended into the realm of de novo design of proteins [36]. Parallel (α/β)_8_—TIM barrel [37,38] served as an exemplary early model system, wherein, the specificity in side-chain packing as well as the pattern of hydrophobicities, both were detected to play their part. However, from all such studies, it was unmistakable that conformational plasticity is an inherent feature in proteins, resulting in structural relaxation to reduce the effect of mutations, particularly applicable in the context of multiple core mutations (Figure 2) in foldable globules [39], which is also relevant for IDPs [40,41]. 

## 3. Mutations in IDPs as Compared to Globular and Membrane Proteins

Although one of the hallmarks of IDPs is to harbor a high degree of structural plasticity, this may not always guarantee compensation of the deleterious effects caused by certain missense mutations. We should recall that many human diseases, such as cancer, diabetes, neurodegenerative and cardiovascular disorders, are associated with IDPs. Interestingly, similar mutational prototypes have generally been found to be more damaging in IDPs than in globular proteins [42,43,44], which is somewhat paradoxical, given the fact that IDPs have a substantially greater degree of structural plasticity and, therefore, are expected to have a corresponding greater potential to compensate for the mutational damage than that of globular proteins. However, the step-wise molecular and cellular consequences of a certain mutational prototype is hierarchical, multi-factored and complex. For example, amyloidosis may be defined as the formation of amyloid fibrils in protein polymers consisting of identical monomeric units which are the macromolecular end-effects responsible for many neurodegenerative disorders (e.g., Parkison’s, Alzheimer’s) which, in turn, is a consequence of β-aggregation. Again, β-aggregation may be accounted for by hydrophobicity and/or β-sheet propensity of a protein region [43]. Comparative studies in α- and γ-synuclein have revealed increased aggregation in the former with a higher propensity for β-sheets, which further suggests that an increased α-helical propensity in the amyloid-forming region may protect against γ-synuclein aggregation [45]. Interestingly, IDPs have been found to be more prone to amyloidosis in spite of having a much lower aggregation propensity (having only one third of aggregation nucleating regions) compared to globular (and membrane) proteins [43]. This high aggregation propensity also explains the considerable amount of structural frustration in globular proteins [43]. However, it may not be straightforward to draw a correlation between the solution conformations of amyloidogenic proteins and their pathogenicity. Though, lately, biophysical characterization of misfolded states and their aggregation mechanisms have gained considerable attention [46], aggregation pathways remain complex (whether pathogenic or not), often involving peculiarities of protein misfolding and characterized by remarkable polymorphism, wherein, the final product may consist of soluble oligomers, fibrils as well as amorphous aggregates [47]. 

**Figure 3:**
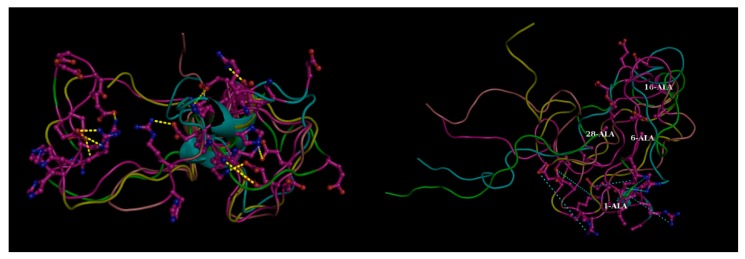
Effect of mutations in disordered proteins. Four transient (flitting) salt bridge forming charged residues (1-Asp, 6-His, 16-Lys, 28-Lys) mutated to alanine in beta amyloid (Aβ42) resulting in the dismantling in salt-bridges globally throughout the structural ensemble (Left Panel: Mutant compared to the Right: Native). These transient salt-bridges continuously keep altering their partners throughout the whole simulation trajectory supporting different conformations at different time points and thereby supporting a conformational ensemble (illustrated in Figure 3. of Reference [19]). The yellow dashed lines in the left panel (native) show the salt-bridges found individually in the five randomly chosen conformers (within 4 Å) while the same connections are shown by thinner cyan dashed lines in the right panel to portray the absence of these ionic interactions (far greater than 4 Å). Molecular Dynamics simulation trajectories collected from Reference [19]. Briefly, explicit-water molecular dynamic (MD) simulation was performed with AMBER 12 at T = 300 K using the ff99SB force field with periodic boundary conditions and TIP3P water model. Figure reconstructed in Pymol.

As a matter of fact, the ‘disease-associated missense mutations’ in IDPs are also found in a higher prevalence with greater functional impact [48] than the ‘neutral polymorphisms’ [49]. More importantly, the IDP-disease-mutations are found to be associated with the ‘disorder-to-order transitions’ [41] at a far greater frequency than the polymorphic ones [46]. The cancerous mutations in p53 [48,50] in its DNA-binding domain are classic examples of IDP-disease-mutations, wherein, dramatic destabilization of the domain renders it disordered at physiological conditions [51]. Overall, there are many investigations associated with mutational studies on IDPs revealing their molecular evolution [27] and pathological features [20]. Traditionally, the mutations can be viewed as mostly ‘hereditary’ [20], chosen on the basis of geographic and ethnic variations, pedigree of individual families with a history of a certain (say, the Alzheimer’s) disease. At the molecular level, one of the major insights revealed by these mutational studies has been the influential role of salt-bridges in mediating the ‘mutation-induced rigidity’ associated with enhanced aggregation of the candidate IDP, which has also found support by recent molecular dynamic (MD) studies exploring the nitty-gritty and transient nature of salt-bridge dynamics (Figure 3) in IDPs [19]. 

Another effective and important way to classify mutations may be based on the actual consequence of a mutation as to whether it purely disrupts the structural integrity of a protein [52] or affects protein functions. For example, proximal residues may co-evolve together in a protein fold to preserve global stability, while point mutations (including insertion–deletions) may potentially fine-tune protein function, modifying functional sites and protein interactions [53]. Again, functional mutations may be proximal or direct to the catalytic/active site [54,55] as well as distal (allosteric and regulatory). The effect of distal mutations have been found to propagate throughout the whole protein fold affecting both its dynamics and catalysis, wherein low-frequency torsional oscillations [56,57] appear to play a pivotal role. Mutational hot-spots [58] have been identified (e.g., in human monoacylglycerol lipase, human DNA polymerase β) based on such long-range communication hubs in protein conformational dynamics [59,60,61]. Such information may also potentially facilitate developing novel ligands with therapeutic value [59]. 

## 4. Probing the Role of Mutations in Diseases: Tracking Changes in Thermodynamic Parameters

Changes in folding and binding free energies (∆∆G) are the standard thermodynamic measures to probe the effect of mutations on protein stability and binding [50]. It has been demonstrated that for assessing the effect, one needs to take into account the relative change in ∆∆G with respect to the ∆G_WT_ rather than considering ∆∆G alone [62]. Changes in ∆∆G were used to characterize sequence and structural patterns on human disease-causing amino acid variants [63,64]. Particular attention was paid to mutations involving a reversal of biophysical characteristics of the wild type residue(s). For example, salt-bridge mutations have been found to be typically disease-causing as demonstrated in the case of hyper-aldosteronism, wherein the mere removal of the charge (while keeping intact the side chain geometry) on a single strategic amino acid site (Glu → Gln) [65], and thereby effectively dismantling a critical salt-bridge, was found to be nitpicking. Salt-bridge mutations in IDPs have also been found to be deleterious with enhanced aggregation of the proteins (e.g., in Alzheimer’s and Parkinson’s Diseases) [20]. Recent MD simulation studies on IDPs have explored a plausible interpretation of the corresponding molecular events, wherein a considerable reduction in the conformational variation was found in Aβ_42_ upon dismantling both high persistence as well as transient salt-bridges [19]. 

Several computational approaches have been developed to predict folding and binding free energy changes (∆∆G) as a mean to link them with pathogenicity of mutations. These approaches vary from sequence-based [66,67], to structure-based [68,69,70], depending on the input [63,64]. Methodologies vary from empirical approaches [71,72], first-principle approaches [73], combination of knowledge-based terms and physics [74,75,76] to machine learning approaches [77,78]. It should be emphasized that for effective drug discovery, one needs to know not only the thermodynamic effects of mutation but also the 3D structure of the target biomolecule(s). Notably, for amyoidogenic proteins, kinetics may take over thermodynamics as aggregation is often found to be kinetically driven [79]. It is also important to consider that a protein is never isolated in a cellular context, rather, all cellular biochemical processes take place in heterogeneous, highly volume occupied, crowded environments [80,81], wherein stabilization of a particular protein [82] may occur by complex formation with specific partner molecules. Taking this into account, most biophysical experimental assays, as well as computational methods, may be seen as reductionist approaches, wherein free energy calculations may strongly be biased and would, therefore, require corrections by appropriate normalization factors [83], also taking into account convergence and sampling [84]. It is, therefore, of utmost importance to, at least, perform cross-validations of the thermodynamic parameters calculated between experimental (calorimetric and/or other indirect spectroscopic techniques) and structure driven computational approaches [85] wherever applicable and possible. In addition, to that end, there have been studies vividly addressing the thermodynamic consequences of excluded volume and macromolecular crowding, both, in vitro and in vivo using labeled tracer macromolecules [80]. Strategies have also been proposed to extend quantitative analyses of crowding from simple model systems to systems with increasing complexity up to the labels of intact cells [80].

## 5. Statistical Classification of Mutations Based on Their Degree of Harmfulness

The effect of some mutations is more pronounced [86] than others. To that end, statistical studies [64,87] have broadly classified the nsSNPs into two major categories: (i) polymorphic (or harmless) and (ii) disease variants. The influential causal factors considered in such statistical studies are genetic variations, frequency of occurrence, and statistical measure(s) of the degree of harmfulness [64,88]. The object of the exercise was to find empirical correlations between the variation type and the degree of harmfulness, if any. To that end, the entire combinatorial space of 380 possible amino acid mutations (20 amino acids each can be replaced by one out of the other 19 makes it 380) (that can occur from a set of 20 naturally occurring amino acids) was explored, and the frequency of each mutation in the corresponding database was recorded. To overcome any possible database-bias, the calculations were repeated as a means to cross-validate the results on updated database(s). Major observations were that in the HumVar dataset [89], 108 out of 380 possible mutations were never found, while, contrastingly, the top 26 most frequent variants made up as much as 46% of the whole dataset. As a matter of fact, only about one quarter (only 87 out of 380) of the variants were found to belong to the “harmless” category [64] in the same database. As a cross-validation, when the analysis was repeated in an expanded dataset of more than three-fold increased size, a jump was observed in the ‘polymorphic-to-disease variant’ ratio from 0.74 to 1.54 in the new compared to the older dataset. Such indifference resulting from database bias inherent to all these statistical/knowledge-based approaches actually speak in favor of using ∆∆G as a more reliable and preferred probe to be applied on a case-to-case basis to make predictions about the effect of a particular mutation in relation to pathogenicity. 

## 6. Mitigating and Clustering the Effects of Disease-Causing Genetic Variants in Relation to Drug Design

With the rapid development of computer techniques, computer-aided approaches have been widely applied in aiding early-stage drug discovery both in industrial as well as in academic projects [90,91,92,93]. By discovering the potential compounds that target and affect the function of specific proteins, biological processes can be modulated to mitigate or eliminate the disease-causing effects [90,92]. Advances in human genome projects have provided a large plethora of target proteins for drug discovery projects [94,95]. Meanwhile, breakthroughs in structural biology have offered in-depth structural information of more and more targets and elucidated the disease mechanisms at the molecular level [96,97,98,99]. Such advances have further stimulated the application of computational approaches to integrate the available structural information, functional mechanism, and physico–chemical properties related to drug discovery [91,100]. Drug discovery traditionally is a time- and energy-consuming process and it would be difficult to imagine that the process can be reduced to the time-span of (say) cancer illness of a single patient. Then again, discovery of compounds to mitigate or eliminate the disease-causing effects induced by a specific amino acid mutation is the main goal of personalized medicines [101]. In other words, benefiting from an individual’s genomic information (by means of comparing to the sequence consensus of the standard human genome), followed by the identification of drug-like compounds such as screening of FDA approved drugs over a particular novel mutation may potentially provide precise treatment to target specific disease-associated mutations on these individuals. In addition, the individual’s genomic information can be of great help to include or exclude patients most appropriate for clinical trails at the final stage of drug development, which would not only increase the safety of the patients but also accelerate the drug testing process. 

In a drug-design methodology, targeting specific disease-causing mutations and elucidation of the mutational effects together is of great importance, especially for the approaches requiring structural information of the target protein. Free energy calculation methods are used to determine the dominant effects of mutations, whether affecting protein stability, protein binding or both. With the in-depth analysis of the effect of mutations at the molecular-level, the disease-causing mutations in the target proteins can further be clustered by their major effects such as destabilizing mutation, catalytic mutations, mutations affecting dimerization or protein conformations [102,103,104]. Such types of classification can help designing drugs for certain groups of mutations with similar effects and is, thus, applicable to a broader spectrum of diagnosis and therapy. 

## 7. Structure-Based Approach in Drug Design

**Figure 4:**
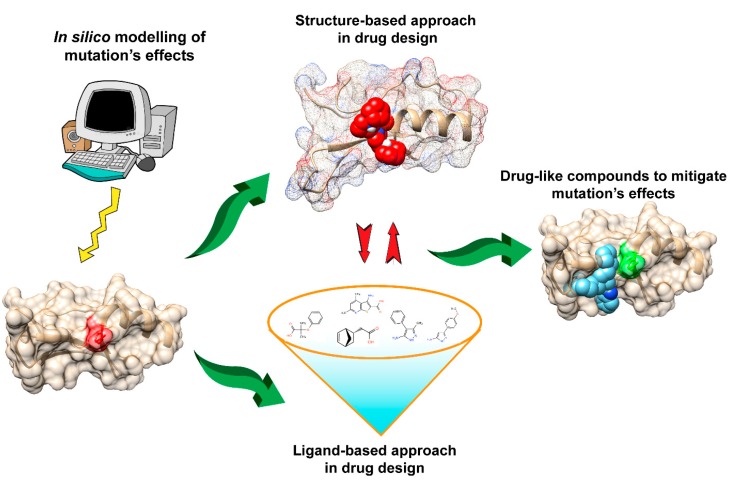
Schematic presentation of the drug discovery process to mitigate the effects of disease-causing mutations.

Structure-based drug design (SBDD) is the computational approach that relies on knowledge of 3D structures (Figure 4) of the biological targets to identify or design the potential chemical structure suitable for clinical tests [100,105]. With the explosion of genomic, functional, and structural information in recent decades, the majority of biological targets with 3D structure have been identified and stimulated the applications of structure-based approaches in the current design pipeline. SBDD is popular for virtual screening to filter the drug-like compounds from a large library of small molecules, including widely applied approaches, such as docking and structure-based pharmacophore design [105]. While the established high-throughput screening (HTS) [106] allows for automatic testing of a wide range of compounds (up to millions), the low success rate and high cost together limit its applications. Alternatively, one can use computational approaches to reduce the number of compounds subjected to testing [105,106], wherein docking and structure-based pharmacophore design are the two most popular approaches, targeting deleterious mutations.

### 7.1. Docking

**Figure 5:**
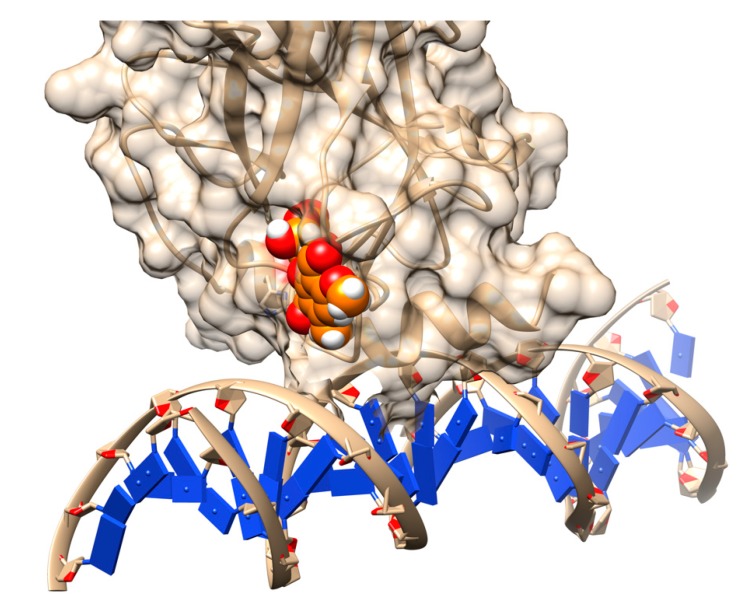
Rescuing the activity of mutant p53 by binding stictic acid into the open L1/L3 pocket. The representative scheme is generated using the structure of p53 core domain complex with DNA (PDB: 1TSR). Atoms in sphere representation belong to stictic acid in a given docked pose (hetero atoms colored as per the default coloring scheme of Chimera). The DNA bases are represented as blue squares.

Docking is one of the most common approaches for compound screening, and the basic idea is to use scoring functions to evaluate the fitness of the target protein in complex with the docked compound [92]. Currently, vast docking programs have been developed to perform fast docking calculations with a wide array of protocols and scoring functions, such as Dock6 [107], Autodock Vina [108], Glide [109], Surflex [110], and many others. Such approaches require the structure of the target protein to be either experimentally solved or computationally modeled. As mentioned above, one should do intensive modeling to generate the best representative structure or set of structures to be subjected to docking [91,111]. In the past, SBDD has been widely applied in mitigating the effects of mutations related to many common diseases. Examples include the p53 protein, which is the so-called “guardian protein” in cancer, functioning as a tumor-suppressor [112]. Again, only, some mutations in p53 result in the malfunctioning of the protein and increase the risk of cancers [113]. In cancer patients, mutations destabilizing the DNA binding to p53 are frequently observed and rescuing the native function(s) in the ‘mutant p53 protein’ is one central objective in current cancer research [114,115]. In the past, it has been shown that the binding of small molecules can stabilize the DNA binding domain and rescue mutant functions [110]. Recent work modeled the wild-type and several mutants [116] to elucidate the mechanism of p53 reactivation [116]. A novel transiently open L1/L3 pocket was identified and indicated the exposure of Cys-124 in the formation of such cavity [116]. Such finding is crucial as Cys-124 has been suggested to be the covalent docking site for known alkylating p53 stabilizers [117] while compounds can be docked onto this pocket to search for other potential stabilizers. As a matter of fact, 1,324 compounds from the NCI/DTP Open Chemical Repository Diversity Set II were docked onto the generated ensemble structures of R273H cancer mutant out of which 45 compounds were selected for biological assay [116]. Finally, one compound, stictic acid (NSC-87511) (Figure 5) was experimentally validated to be an efficient reactivation compound for mutant p53 [116].

Besides cancer research, docking based screening has also been used in rare diseases. Snyder–Robinson Syndrome (SRS) is a rare X-linked mental disease, caused by the malfunctioning of an important human enzyme, the spermine synthase [118]. Spermine synthase functions as homo-dimer and mutations affecting the dimerization such as G56S are shown to abolish the enzyme activity to result in the disease [96,118]. Recent work has targeted identification of dimer stabilizers by binding to the mutant homo-dimer interface [119]. Integrated large commercial compound libraries were used for this docking-based virtual screening with the representative structures of the dimer [119]. The best-ranked 51 compounds were then subjected to experimental screening out of which three top-ranked compounds (also known as ‘leads’) have been shown to enhance the catalytic activity up to 30% [119,120]. 

### 7.2. Structure-Based Pharmacophore Design

Pharmacophore models can be used to make an ensemble of abstract steric and electronic features representing macromolecular (target protein) interactions with drug-like small molecules [121,122]. In other words, three-dimensional arrangements of these features such as hydrophobic centroids, aromatic rings and hydrogen bonds are representation of the binding mode between the ligand and the target [122,123]. Pharmacophores are generated from common features of active ligands, which are identified by aligning or superimposing the conformers of either ligand-target complexes or known active molecules [123]. Multiple degenerate atomic models can potentially be output from pharmacophore modeling programs requiring further optimization and validation to select the best one. Pharmacophore models are commonly used for virtual screening of active small molecules from large compound databases [121,122,123]. Such approaches can be more efficient than docking for certain targets, especially when a large number of existing known active compounds are available [124,125,126]. 

Pharmacophore models have also been used to identify active molecules to mitigate the effects of mutations in many diseases [108,109,110,111]. For cases where a sufficient number of active molecules are previously known for generating high-quality pharmacophore models, pharmacophore proves to be a powerful tool for drug ‘lead’ identification [106]. Recent work has applied structure-based pharmacophore analysis to identify the novel *ROS-1* inhibitors to curb the drug resistance problem caused by mutations [127]. Proto-oncogene receptor tyrosine kinase ROS-1 is ectopicly and oncogenicly expressed in many cancers, mainly in non-small cell lung cancer (NSCLC) [127]. ROS-1 is highly homologous with the kinase domain of anaplastic lymphoma kinase (ALK) and FDA approved ALK inhibitors such as Crizotinib are experimentally validated as therapeutics against *ROS-1* driven tumors [127]. However, these commercial *ROS-1* inhibitors lack a broad spectrum of activity due to the growing resistance from ROS-1 mutations, primarily G2032R [128]. Following on, a pharmacophore model was built using the complex structure of both wildtype (WT) and mutant ROS-1 with previously known inhibitors to identify more general inhibitors against both WT and mutant [129,130]. Pharmacophore-based virtual screening was then performed to selected candidates from commercial databases with further filtering and scoring analysis. Five hits were eventually identified with good binding affinities to both WT and mutant [130].

Thus, pharmacophore essentially defines the interaction framework among the active ligands, and their specific targets [121,122] and the corresponding models can also be built with libraries of active ligands alone, in the absence of the 3D structure of the target—an approach known as ligand based pharmacophore. The models, therein, can then be trained for discrimination between active and inactive molecules [121]. In fact, this serves as the prime reason of widespread use of pharmacophore models in virtual screening especially when lacking the target structure. In addition, as the pharmacophore model represents the binding (or interaction map) of ‘active compounds-target interaction’, it provides a plausible relationship between the structure and the ligand activity and could help to elucidate the underlying biochemical mechanism to further guide the design of the novel active compounds [122]. For example, by exploring the different pharmacological properties, recent studies have seemed to improve the potency of existing pharmacophore and designed novel epidermal growth factor receptor (EGFR) inhibitor potentially inhibited by primary mutants (L858R, del9) and drug-resistant mutants, such as L858R/T790M [128].

## 8. Ligand-Based Approaches in Drug Design

In the lack of structural information of the target protein(s), the aforementioned structure-based approaches may not be suitable for drug design. As an alternative, ligand-based drug design (LBDD) can be applied to aid such cases [131,132,133,134]. Ligand-based methods only focus on the analysis of physico–chemical properties of known ligands that interact with the target of interests. Most popular approaches, however, are the quantitative structure–activity relationship (QSAR) models and the ligand-based pharmacophore modeling [134]. In terms of drug design, targeting the mutant proteins, LBDD could be efficient for novel mutations whose effects have not yet been investigated.

The basic assumption in ligand-based drug design is that small molecules with similar shape and biophysical properties will likewise interact with the same target receptor [123,131]. By identifying the fingerprints of known active ligands and constructing LBDD models, large databases can be screened to retrieve the novel compounds as potential leads for the target of interest [134]. QSAR is a widely applied LBDD approach, which utilizes mathematical models to correlate the physio–chemical properties of compounds to their experimentally measured bio-activity. Generally, QSAR methodology identifies the molecular descriptors associated with properties of the ligands and further uses mathematical models to discover correlations between the molecular descriptors and their biological activity. Finally, these QSAR models are tested and validated for the predicted biological activity of the compounds. As it stands, the current state-of-the-art is to apply the QSAR models widely in computer-aided drug design, targeting the mutant protein(s). One major success is the discovery of the potential corrector for cystic fibrosis (CF) mutations, namely, F508del in cystic fibrosis transmembrane conductance regulator gene (CFTR) [132,133,134]. F508del is the most frequent CF causing mutation, which leads to the improper folding of the protein and its degradation [132]. Subsequent to the identification, QSAR analysis has further been applied to guide the synthesis of novel compounds to treat CF by improving the trafficking of the mutant CFTR (the CF corrector) [133]. Recent works have collected all compounds known to improve the F508del trafficking and then applied QSAR analysis to decipher the critical chemical descriptors for the potential F508del correctors [133]. A novel predictive model was then constructed with these descriptors to provide further guidelines to the design and optimization of the novel corrector [134]. Again, the combination of ligand and structure-based approaches is expected to add significantly more to the current state-of-the-art [135,136]. Such combinations can either be sequential, parallel or hybrid, integrated contextually into a drug discovery pipeline, and, have already shown much promise [136]. 

A more sophisticated case would be to consider targeting proteins that lack both 3D structures and known active ligands and, therefore, will not have sufficient information to build robust pharmacophore and QSAR models. On such instances, one may switch on to sequence-based ligand predictor approaches such as meta-structure [137]. The basic idea behind developing meta-structure is the transformation of 3D structural information into the topological space via calculating the residue interaction networks from a database [137,138]. The residues and the corresponding neighborhood relationships are represented by nodes and edges. The predictor is trained against sets of representative protein 3D structure to derive statistical topological information for all possible amino acid pairs and, thus, can be subsequently used to perform predictions solely based on primary sequences [137]. Based on the sequence analysis, the quantitative information about the local secondary structure and residue compactness for each residue can be acquired to describe the intricate interaction networks in the topological space for the target protein [137]. Such meta-structure features can further be applied in drug development especially for target proteins that lack 3D structural information. For example, inspired by the protein-structure similarity clustering (PSSC) approach in structure-based drug development [139], the meta-structure similarities in ligand binding site can be used to cluster proteins with similar ligand-binding properties. Thus, the meta-structure features of any one member of the cluster would serve as a valuable starting point for ligand development of other members in the cluster [137]. 

## 9. Aiding Drug Design by the Knowledge of Mutations on Globular, Membrane and Disordered Proteins

Understanding of molecular mechanism of disease-associated mutations can directly be applied to drug design [91,101]. Structural biology has been instrumental in such understanding, effectively contributing to early drug discovery [140], and, also elucidating the impacts of disease-associated mutations and drug resistance in cancers and infectious diseases. Information regarding the differential effects of mutations on globular, membrane and disordered proteins may serve beneficially to select and apply the most appropriate and effective strategy to design potential drug-like molecules for each individual case. The majority of diseases are directly associated with the alterations of binding stability or folding stability of mutated proteins [63]—probed by binding or folding free energies. Such information also indicates to what extent the mutations are disrupting the protein interactions or structural integrity providing important guidelines towards the design of stabilizers and/or inhibitors to mitigate or eliminate the deleterious effects of mutations. 

In addition, having 3D structures of target proteins is of great advantage to be used in free energy calculations coupled with MD simulations to extensively investigate the underlying structural mechanism (e.g., disruption of the hydrophobic core or loss of hydrogen bonding) of the mutational effects on binding or folding. As discussed in an earlier section, such information has been successfully used to identify the correct drug-like molecules targeting the mutations related to Snyder–Robinson Syndrome (SRS). SRS is caused by the malfunctioning of the human enzyme, spermine synthase (a globular protein), wherein the known existing deleterious mutations affect the native protein functionality by a wide range of molecular mechanisms, such as dedimerization, destabilization of the monomer, and disruption of the catalytic core [96,119,120,141]. 

Mutational resistance towards drugs also limits the lifetime of many successful drugs. As an alternative to the design of novel drug-like molecules to overcome such resistance, strategies, such as ensemble-based protein design [142], have been developed to be administered early in the development process to predict and overcome the effects of possible mutational resistance (e.g., in dihydrofolate reductase of *Staphylococcus aureus)*. Such design protocol has a dual attribute, namely, positive design to maintain catalytic function and negative design to interfere with binding of a lead inhibitor simultaneously. 

Alteration in protein conformation and dynamics are also closely related to a significant number of human diseases [143,144,145]. Computational approaches, such as MD or Mote Carlo (MC) simulations, are powerful tools to study protein dynamics. Mutations can alter protein dynamics in various ways, such as altering local flexibility, transition in conformational states, allosteric regulations etc. Exploring allosteric regulations may serve as potential alternatives for the cases where the native binding pocket is deemed to be too difficult to bind with small molecules [91]. Solving experimental structures of destabilizing mutants is often found difficult, particularly for membrane proteins due to their inherent insolubility and instability [146,147] and in such cases, molecular modeling of mutant structures can give some guidelines about the mutational effects. Such alternative structures are frequently subjected to docking of compound libraries in virtual screening—a methodology known as “ensemble-based drug design” [148,149]. Especially for IDPs where there is a definite lack of ordered structures, molecular modeling and MD simulation have together been widely applied to retrieve the representative structural ensemble in structure-based drug design [145,150]. 

On the other hand, high-throughput screening and rational drug design have considerably aided drugging membrane protein interactions as they are accessible on the cell surface and can directly alter cellular signaling [151]. This, in fact, is the key reason why the majority of therapeutics target membrane proteins. Techniques, such as alanine scanning, have also served to identify stabilizing mutations in the computational design of membrane proteins as well as in drug development [152]. To that end, frameworks, such as RosettaMP, have been developed to provide a general membrane representation that interfaces with scoring, conformational sampling, and mutation routines offering great ease and flexibility to integrate them into new design protocols [153]. Peptide architectonics [154] have been a relatively new addition in the subject, wherein, the idea is to select for sub-sequences of a native peptide, selectively toxic towards the pathogenic membrane proteins alone. As an alternative to drugging (as there is often a lack of structural information for transmembrane proteins), engineering of protein therapeutics [147] has also been attempted to membrane protein targets, though, its full potential is yet to be explored. 

## 10. Conclusions

Macromolecular structural analyses may potentially be used to aid probing of genetic variants linked with disease. Such studies are usually complemented by a wide range of biophysical solution assays and computational modeling. Research along these directions has also opened up avenues towards developing diagnostic tools and plausible therapeutics. In such a context, it is of foremost importance to conceptualize (i) how traditionally mutational effects on protein stability and binding have been probed and (ii) the basis of the differential effects of mutations to different classes of proteins (globular, membrane, and disordered proteins) based on conformational relaxation, structural plasticity, compensation, and other physico–chemical factors. In the second half of the paper, we took the opportunity to discuss how this knowledge-base of the effect of mutations in globular, membrane, and disordered proteins may potentially aid drug design. As a probing technique, we particularly highlighted the importance of tracking changes in thermodynamic parameters (∆G_WT_) and also took the opportunity to discuss the limitations of knowledge-based approaches such as the statistical classification of mutations based on their degree of harmfulness. The review particularly highlighted the emergence of the ever-so-promising recent approach to computationally mitigate the effects of disease-causing genetic variants, alternative to the traditional approaches in designing inhibitors. A wide array of structure-based approaches in drug design including docking, structure-based pharmacophore design, and ligand-based approaches have been vividly discussed along with their proper context of applicability, as to whether they are to be aided in presence or absence of the experimental coordinates of the target protein and/or known ligands.
